# Coupled VAE: Improved Accuracy and Robustness of a Variational Autoencoder

**DOI:** 10.3390/e24030423

**Published:** 2022-03-18

**Authors:** Shichen Cao, Jingjing Li, Kenric P. Nelson, Mark A. Kon

**Affiliations:** 1 Worcester Polytechnic Institute, Worcester, MA 01609, USA; cao.schen@gmail.com; 2 Mathematics & Statistics Department, Boston University, Boston, MA 02215, USA; jli0203@bu.edu (J.L.); mkon@bu.edu (M.A.K.); 3 Photrek, Watertown, MA 02472, USA

**Keywords:** machine learning, entropy, robustness, statistical mechanics, complex systems

## Abstract

We present a coupled variational autoencoder (VAE) method, which improves the accuracy and robustness of the model representation of handwritten numeral images. The improvement is measured in both increasing the likelihood of the reconstructed images and in reducing divergence between the posterior and a prior latent distribution. The new method weighs outlier samples with a higher penalty by generalizing the original evidence lower bound function using a coupled entropy function based on the principles of nonlinear statistical coupling. We evaluated the performance of the coupled VAE model using the Modified National Institute of Standards and Technology (MNIST) dataset and its corrupted modification C-MNIST. Histograms of the likelihood that the reconstruction matches the original image show that the coupled VAE improves the reconstruction and this improvement is more substantial when seeded with corrupted images. All five corruptions evaluated showed improvement. For instance, with the Gaussian corruption seed the accuracy improves by $10^{14}$ (from $10^{-57.2}$ to $10^{-42.9}$) and robustness improves by $10^{22}$ (from $10^{-109.2}$ to $10^{-87.0}$). Furthermore, the divergence between the posterior and prior distribution of the latent distribution is reduced. Thus, in contrast to the β-VAE design, the coupled VAE algorithm improves model representation, rather than trading off the performance of the reconstruction and latent distribution divergence.

## 1. Introduction

An overarching challenge in machine learning is the development of methodologies that ensure the accuracy and robustness of models given limited training data. By accuracy, we refer to the metrics of information theory, such as minimizing the cross-entropy or divergence of an algorithm. In this paper, we define a measure of robustness based on a generalization of information theory. The variational autoencoder (VAE) contributes to improved learning of models by utilizing approximate variational inference [1,2]. By storing a statistical model rather than a deterministic model at the latent layer, the algorithm has increased flexibility in its use for reconstruction and other applications. The variational inference is optimized by minimization of a loss function, the so-called negative evidence lower bound, which has two components. The first component is a cross-entropy between the generated and the source data, also known as the expected negative log-likelihood, while the second is a divergence between the prior and the posterior distributions of the latent layer.

Our goal in this research is to provide an evaluation as to whether a generalization of information theory can be applied to improving the robustness of machine learning algorithms. Robustness of autoencoders to outliers is critical for generating a reliable representation of particular data types in the encoded space when using corrupted training data [3]. In this paper, a generalized entropy function is used to modify the negative evidence lower bound loss function of a variational autoencoder. With the MNIST handwritten numerals dataset, we are able to measure the improvement in the robustness of the reconstruction, using a metric also derived from the generalization of information theory. In addition, we find that the accuracy of the reconstruction, as measured by Shannon information theory, is also improved. Furthermore, the divergence between the latent distribution posterior and prior is also reduced. This is important to ensure that the reconstruction improvement is not a result of degrading the latent layer.

Our study builds from the work of Kingma and Welling [4] on variational autoencoders and Tran et al. [5] on deep probabilistic programming. Variational autoencoders are an unsupervised learning method for training encoder and decoder neural networks. Between the encoder and decoder, the parameters of a multidimensional distribution are learned to form a compressed latent representation of the training data [6]. It is an effective method for generating complex datasets such as images and speech. Zalger [7] implemented the application of VAE for aircraft turbomachinery design and Xu et al. [8] used VAEs to achieve unsupervised anomaly detection for seasonal key performance indicators (KPIs) in web applications. VAEs have been used to construct probabilistic models of complex physical phenomena [9]. Autoencoders can use a variety of latent variable models, but restricting the models can enhance performance. Sparse autoencoders add a penalty for the number of active hidden layer nodes used in the model. Variational autoencoders further restrict the model to a probability distribution $q_{\phi }\left(z|x\right)$ specified by a set of encoder parameters ϕ which approximates the actual conditional probability $p\left(z|x\right)$. Variational inference, as reviewed by Blei et al. [10], is used to learn this approximation by minimizing an objective function such as the Kullback–Liebler divergence. The decoder learns a set of parameters θ for a generative distribution $q_{\theta }\left(x^{\prime }|z\right)$, where z is the latent variable, and $x^{\prime }$ is the output generated data. The complexity of the data distribution p(x) makes direct computation of the divergence between the approximate and exact latent conditional probabilities intractable; however, a variational or evidence lower bound (ELBO) is computable and consists of two components, the expected reconstruction log-likelihood of the generated data (cross-entropy) and the negative of the divergence between the latent posterior conditional probability $q_{\phi }\left(z|x\right)$ and a latent prior distribution p(z), which is typically a standard normal distribution but can be more sophisticated for particular model requirements.

Recently, Higgins et al. [11] proposed a β-VAE framework, which can provide a more disentangled latent representation z [12] by increasing the weight of the KL-divergence term of the ELBO. Since the KL-divergence is a regularization that constrains the capacity of the latent information channel z, increasing the weight of the regularization with β>1 puts pressure on the learnt posterior so it is more tightly packed. The effect seems to be an encouragement of each dimension to store distinct information and excess dimensions as highly packed noise. However, this improvement is a trade-off between the divergence and reconstruction components of the ELBO metric. We will show that the coupled VAE algorithm improves both components of the ELBO.

The next section provides an introduction to the design of the variational autoencoder. A comparison with other generative algorithms is included. Section 3 introduces nonlinear statistical coupling and its application to defining metrics for the robustness, accuracy, and decisiveness of decision algorithms. In this paper, use of the uppercase letter for the terms ’Robustness’, ’Accuracy’, and ’Decisiveness’ refers to the specific metrics, which will be introduced in Section 3.1. Lowercase letters for these terms will be used when referring to the general properties. Following the definition of the reconstruction assessment metrics, the generalization of the negative ELBO is defined. This coupled negative ELBO provides control over the weighting of rare versus common samples in the distribution of the training set. Additional details of the derivation of the generalized negative ELBO function and metrics are provided in Appendixes Appendix A.1 and Appendix A.2, respectively. In Section 4, the improved autoencoder is evaluated using the MNIST handwritten numeral test set. Measurements of the reconstruction and the characteristics of the posterior latent variables are analyzed. Section 5 provides a visualization of the changes in the latent distribution using a 2-dimensional distribution. Section 6 demonstrates that the coupled VAE algorithm provides significantly improved stability in the model performance when the input image is corrupted from the training set. This provides evidence of the improved robustness of the algorithm. Section 7 provides a discussion, conclusion, and suggestions for future research.

## 2. The Variational Autoencoder

**Figure 1:**
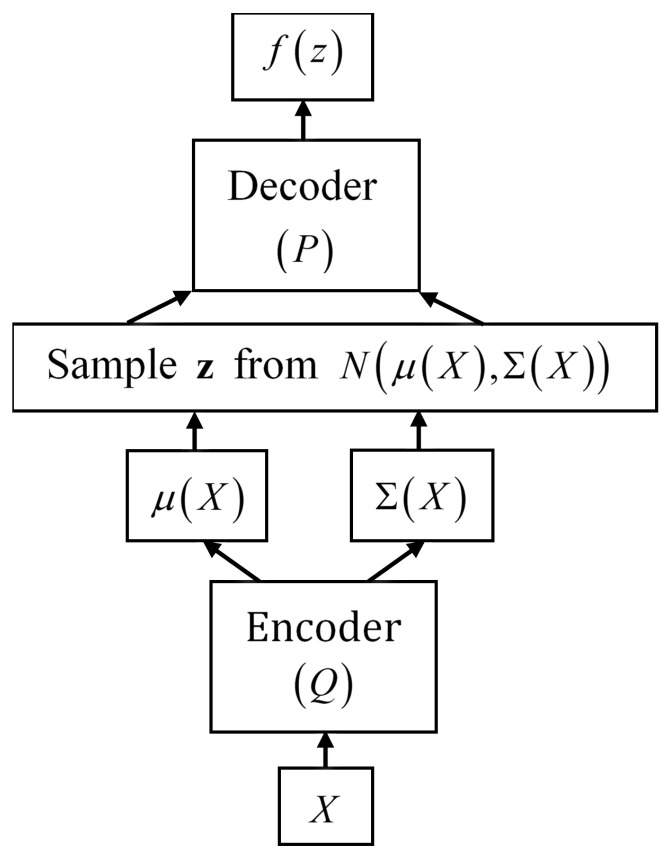
The variational autoencoder consists of an encoder, a probability model, and a decoder.

A variational autoencoder consists of an encoder, a decoder, and a loss function. Figure 1 represents the basic structure of an autoencoder. The encoder *Q* is a neural network that converts high-dimensional information from the input data into a low-dimensional hidden, latent representation z. Some information is lost during this data compression because the dimension is reduced. The decoder *P* decompresses from latent space z to reconstruct the data. While, in general, autoencoders can learn a variety of representations, VAEs especially learn the parameters of a probability distribution. The model used here learns the means and standard deviations θ of a collection of multivariate Gaussian distributions and stores this information in a two-layered space. The training loss function, which is the negative evidence lower bound, is optimized by using stochastic gradient descent.

### 2.1. Vae Loss Function

The encoder reads the input data and compresses and transforms it into a fixed-shape latent representation z, while the decoder decompresses and reconstructs the information from this latent representation, outputting specific distribution parameters to generate a new reconstruction $x^{\prime }$. The true posterior distribution $p(z|x^{\left(i\right)})$ of z given $i^{th}$ datapoint $x^{\left(i\right)}$ is unknown, but we use the Gaussian approximation $q(z|x^{\left(i\right)})$ with mean vector $\mu^{\left(i\right)}$ and covariance matrix $\mathrm{diag}{\left(\sigma_{1}^{2},\cdots ,\sigma_{d}^{2}\right)}^{\left(i\right)}$ instead. The goal of the algorithm is to maximize the variational or evidence lower bound (ELBO) on the marginal density of individual datapoints.

For a dataset $X={\left\{x^{\left(i\right)}\right\}}_{i=1}^{N}$ consisting of *N* independent and identically distributed samples, the variational lower bound for the $i^{th}$ datapoint or image $x^{\left(i\right)}$ in the original VAE algorithm [4] is

(1) $$ELBO\left(x^{\left(i\right)}\right)=-D_{KL}\left(q\left(z|x^{\left(i\right)}\right)\;\|\;p\left(z\right)\right)+E_{q\left(z|x^{\left(i\right)}\right)}\left[\log p\left(x^{\left(i\right)}|z\right)\right].$$

The first term on the right-hand side is the negative Kullback–Leibler divergence between the posterior variational approximation q(z|x) and a prior distribution z which is selected to be a standard Gaussian distribution. The second term on the right-hand side is denoted as the expected reconstruction log-likelihood, and is referred to as the cross-entropy. Let $n_{z}$ be the dimensionality of z; then, the Kullback–Leibler divergence simplifies to

(2) $$\begin{array}{cc} -D_{KL}\left(q\left(z|x^{\left(i\right)}\right)\;||\;p\left(z\right)\right)\;=\; & \int_{}q\left(z|x^{\left(i\right)}\right)\left(\log p\left(z\right)-\log q\left(z|x^{\left(i\right)}\right)\right)dz \end{array}$$

(3) $$\begin{array}{cc} = & \frac{1}{2}\sum_{j=1}^{n_{z}}\left(1+\log\left({\left(\sigma_{j}\right)}^{2}\right)-{\left(\mu_{j}\right)}^{2}-{\left(\sigma_{j}\right)}^{2}\right). \end{array}$$

The expected reconstruction log-likelihood (cross-entropy) $E_{q\left(z|x^{\left(i\right)}\right)}\left[\log p\left(x^{\left(i\right)}|z\right)\right]$ can be estimated by sampling, i.e.,

(4) $$E_{q\left(z|x^{\left(i\right)}\right)}\left[\log p\left(x^{\left(i\right)}|z\right)\right]=\frac{1}{L}\sum_{l=1}^{L}\left(\log p\left(x^{\left(i\right)}|z^{\left(i,l\right)}\right)\right),$$

where *L* denotes the number of samples for each datapoint and we set L=1 in our study. Supposing data x given z has the following probability density,

(5) $$\log p\left(x|z\right)\;=\sum_{i=1}^{n_{x}}\left(x_{i}\log y_{i}\;+\;\left(1-x_{i}\right)\;\log\left(1-y_{i}\right)\right),$$

where y is the output of the decoder. Therefore, the loss function can be calculated by

(6) $$L\left(x^{\left(i\right)}\right)\;=-ELBO\left(x^{\left(i\right)}\right)\;=D_{KL}\left(q\left(z|x^{\left(i\right)}\right)\;\|\;p\left(z\right)\right)\;-\;\frac{1}{L}\sum_{l=1}^{L}\left(\log p\left(x^{\left(i\right)}|z^{\left(i,l\right)}\right)\right).$$

For our work, the loss function is modified to improve the robustness of the variational autoencoder, something that will be discussed in Section 4.

### 2.2. Comparison with Other Generative Machine Learning Methods

The paradigm of generative adversarial networks (GANs) is a recent advance in generative machine learning methods. The basic idea of GANs was published in a 2010 blog post by Niemitalo [13], and the name ‘GAN’ was introduced by Goodfellow et al. [14]. In comparison with variational autoencoders, generative adversarial networks are used for optimizing generative tasks specifically. GANs can produce models with true latent spaces, as is the case of bidirectional GAN (BiGAN) and adversarially learned inference (ALI) [15,16], which are designed to improve the performance of GANs. However, GANs cannot generate reasonable results when data are high-dimensional [17]. By contrast, as a probabilistic model, the specific goal of a variational autoencoder is to marginalize out noninformative variables during the training process. The ability to use complex priors in the latent space enables existing expert knowledge to be incorporated.

Bayesian networks form another generative model. Pearl [18] proposed the Bayesian network paradigm in 1985. Bayesian networks have a strong ability to capture the symbolic figures of input information and combine objective probabilities with subjective estimates for both qualitative and quantitative modeling. The basic concept of Bayesian networks is built on Bayes’s theorem. Another effective way to solve for the posterior of the distribution derived from neural networks is to train and predict using variational inference techniques [19]. Compared with the original Bayesian network, the basic building blocks of deep networks provide multiple loss functions for making multitarget predictions, for transfer learning, and for varying outputs depending on the situation. The improvement of the deeper architectures, using VAE specifically, continues to occur.

Other generative models are now commonly combined with a variational autoencoder to improve performance. Ebbers et al. [20] developed a VAE with a hidden Markov model (HMM) as the latent model for discovering acoustic units. Dilokthanakul et al. [2] studied the use of Gaussian mixture models as the prior distribution of the VAE to perform unsupervised clustering through deep generative models. They showed a heuristic algorithm called ‘minimum information constraint’ and it is capable of improving the unsupervised clustering performance with this model. Srivastava and Sutton [1] presented the effective autoencoding variational Bayes-based inference method for latent Dirichlet allocation (LDA). This model solves the problems caused by autoencoding variational Bayes by the Dirichlet prior and by component collapsing. Additionally, this model matches traditional methods’ inaccuracy with much better inference time.

## 3. Accounting for Risk with Coupled Entropy

Machine learning algorithms, including the VAE, have achieved efficient learning and inference for many image processing applications. Nevertheless, assuring accurate forecasts of the uncertainty is still a challenge. Problems such as outliers and overfitting impact the robustness of scientific prediction and engineering systems. This paper concentrates on assessing and improving the robustness of the VAE algorithm.

In this study, we draw upon the principles of nonlinear statistical coupling (NSC) [21,22] to define a generalization to information theory and apply the resulting entropic functions to the definition of the negative ELBO loss function for the training of the variational autoencoder [23]. NSC is derived from nonextensive statistical mechanics [24], which generalizes the variational calculus of maximum entropy to include constraints related to the nonlinear dynamics of complex systems and in turn to the nonexponential decay of the maximizing distributions. The NSC frame focuses this theory on the role of nonlinear coupling κ in generalizing entropy and its related functions. The approach defines a family of heavy-tailed (positive coupling) and compactly supported (negative coupling) distributions which maximize a generalized entropy function referred to as coupled entropy. The variational methods underlying NSC can be applied to a variety of problems in mathematical physics [25,26]. Here, we examine how NSC can broaden the role of approximate variational inference in machine learning to include sensitivity to the risks of outlier events occurring in the tail of the distribution of the phenomena being learned.

### 3.1. Assessing Probabilistic Forecasts with the Generalized Mean

First, proper metrics are needed to evaluate the accuracy and robustness of machine learning algorithms, such as VAE. The arithmetic mean and the standard deviation are widely used to measure the central tendency and fluctuation, respectively, of a random variable. Nevertheless, these are inappropriate for probabilities, which are formed by ratios. A random variable formed by the ratio of two independent random variables has a central tendency determined by the geometric mean, as described by McAlister [27]. Information theory addresses this issue by taking the logarithm of the probabilities, then the arithmetic mean; however, we will show that the generalizations of information theory are easier to report and visualize in the probability domain.

In [28], a risk profile was introduced, which is the spectrum of the generalized means of probabilities and provides an assessment of the the central tendency and fluctuations of probabilistic inferences. The generalized mean ${\left(\frac{1}{N}\sum_{i=1}^{N}p_{i}^{r}\right)}^{\frac{1}{r}}$ is a translation of generalized information-theoretic metrics back to the probability domain, and is derived in the next section. Its use as a metric for evaluating and training inference algorithms is related to the Wasserstein distance [29], which incorporates the generalized mean. The accuracy of the likelihoods is measured with robust, neutral, and decisive risk bias using the $r=-\frac{2}{3}$, r=0 (geometric) and r=1 (arithmetic) means, respectively. With no risk bias (r=0), the geometric mean is equivalent to transforming the cross-entropy between the forecast $p_{i}$ and the distribution of the test samples to the probability domain. The arithmetic mean (r=1) is a simple measure of the Decisiveness (i.e., were the class probabilities in the right order so that a correct decision can be made?). This measure de-weights probabilities near zero since increasing *r* reduces the influence of small probabilities on the average. To complement the arithmetic mean, we choose a negative conjugate value. The conjugate is not the harmonic mean (r=−1) because this turns out to be too severe a test. Instead, $r=-\frac{2}{3}$ is chosen based on a dual transformation between heavy-tail (positive κ) and compact-support (negative κ) domains of the coupled Gaussian distribution. The risk sensitivity *r* can be decomposed into the nonlinear coupling and the power and dimension of the variable $r\left(\kappa ,\alpha ,d\right)=\frac{-\alpha \kappa }{1+d\kappa }$. The dual transformation between the positive/negative domains of the coupled Gaussians has the following relationship: $\hat{\kappa }\Leftrightarrow \frac{-\kappa }{1+d\kappa }$. Taking α=2 and d=1, the coupling for a risk bias of one is $1=\frac{-2\kappa }{1+\kappa }\Rightarrow \kappa =-\frac{1}{3}$ and the conjugate values are $\hat{\kappa }=\frac{\frac{1}{3}}{1-\frac{1}{3}}=\frac{1}{2}$ and $\hat{r}=\frac{-2\cdot \frac{1}{2}}{1+\frac{1}{2}}=-\frac{2}{3}$ [23]. The Robustness metric increases the weight of probabilities near zero since negative powers invert the probabilities prior to the average.

For simplicity, we refer to these three metrics as the Robustness, Accuracy, and Decisiveness. The label ‘accuracy’ is used for the neutral accuracy, since ‘neutralness’ is not appropriate and ‘neutral’ does not express that this metric is the central tendency of the accuracy. Summarizing:

(7) $$Decisiveness\;\;(\mathrm{arithmetic}\;\mathrm{mean}):\;\;\frac{1}{N}\sum_{i=1}^{N}p_{i}.$$

(8) $$Accuracy\;\;(\mathrm{geometric}\;\mathrm{mean}):\;\;\prod_{i=1}^{N}p_{i}^{\frac{1}{N}}.$$

(9) $$Robustness\;\;(-2/3\;\mathrm{mean}):\;\;{\left(\frac{1}{N}\sum_{i=1}^{N}p_{i}^{-\frac{2}{3}}\right)}^{-\frac{3}{2}}.$$

**Figure 2:**
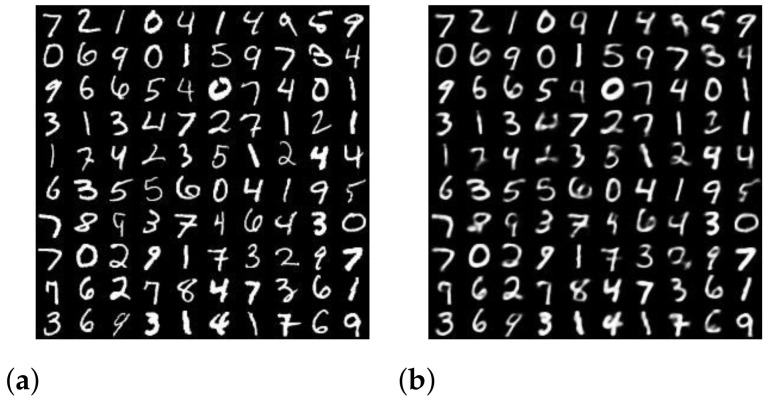
Example set of (**a**) MNIST input images and (**b**) VAE-generated output images.

**Figure 3:**
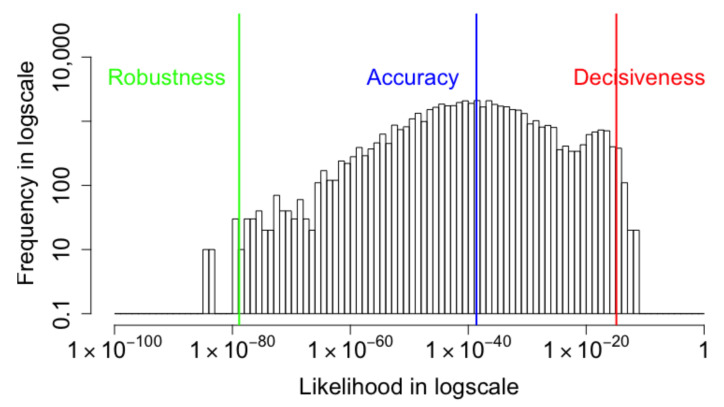
A histogram of the likelihoods that the VAE-reconstructed images match the input images. The objective of the coupled VAE research is to demonstrate that the Robustness, which is the −2/3 generalized mean, can be increased by penalizing the cost of producing outlier reconstructions. The Accuracy is the exponential of the average log-likelihood and the Decisiveness is the arithmetic mean.

Similar to the standard deviation, the arithmetic mean and −2/3 mean play roles as measures of the fluctuation. Figure 2 shows an example of input images from the MNIST dataset and the generated output images produced by the VAE. Despite the blur in some output images, the VAE succeeds in generating very similar images to the input. However, the histogram in Figure 3, which plots the frequency of the likelihoods over a log scale, shows that the probabilities of ground truth range over a large scale. The geometric mean or Accuracy captures the central tendency of the distribution at $10^{-37}$. The Robustness and the Decisiveness capture the span of the fluctuation in the distribution. The −2/3 mean or Robustness is $10^{-77}$ and the arithmetic mean or Decisiveness is $10^{-15}$. The minimal value of the −2/3 mean metric is an indicator of the poor robustness of the VAE model, which can be improved. We measure and display the performance in the probability space in order to simplify the comparison between the three metrics. In the next subsection, we will show their relationship with a generalization of the log-likelihood. If, however, we were to plot histograms in the log-space, separate histograms would be required for each metric. By using the probability space, we can display one histogram overlaid with three different means. Appendix A.2 describes the origin of the Robustness–Accuracy–Decisiveness metrics.

In order to improve performance against the robust metric, the training of the variational autoencoder needs to incorporate this generalized metric. To do so, we derive a coupled loss function in the next subsection.

### 3.2. Definition of Negative Coupled ELBO

As we discussed in Section 2, the goal of a VAE algorithm is to optimize a low-dimensional model of a high-dimensional input dataset. This is accomplished using approximate variational inference by maximizing an evidence lower bound (ELBO). Equivalently, the negative ELBO defines a loss function which can be minimized, $L\left(x^{\left(i\right)}\right)=-ELBO\left(x^{\left(i\right)}\right)$. In this paper, we provide initial evidence that the accuracy and robustness of the variational inference can be improved by generalizing the negative ELBO to account for the risk of outlier events. Here, we provide a definition of the generalization and in Appendix A.1 a derivation is provided.

The generalized loss function in the coupled variational autoencoder (VAE) method is defined as follows.

**Definition** **1.** *(Negative Coupled ELBO). Given the $i^{th}$ datapoint $x^{\left(i\right)}$, the corresponding latent variable value z, and the output value y of the decoder using the Bernoulli distribution, then the loss function for the coupled VAE algorithm is given by*

(10) $$L_{\kappa }\left(x^{\left(i\right)}\right)=D_{\kappa }\left(q\left(z|x^{\left(i\right)}\right)\;\|\;p\left(z\right)\right)+H_{\kappa }\left(x,y\right),$$

*where*

(11) $$\begin{array}{cc} \; & D_{\kappa }\left(q\left(z|x^{\left(i\right)}\right)\|\;p\left(z\right)\right)\\ \equiv & \prod_{j=1}^{n_{z}}\int_{}\frac{{q(z_{j}|x^{\left(i\right)})}^{1+\frac{2\kappa }{1+\kappa }}}{\int_{}q(z_{j}|x^{\left(i\right)}){}^{1+\frac{2\kappa }{1+\kappa }}dz_{j}}\frac{1}{2}\left(\ln_{\kappa }\left(q{\left(z_{j}|x^{\left(i\right)}\right)}^{-\frac{2}{1+\kappa }}\right)-\ln_{\kappa }\left(p{\left(z_{j}\right)}^{-\frac{2}{1+\kappa }}\right)\right)dz_{j}\\ = & \prod_{j=1}^{n_{z}}\frac{1}{2\kappa }\int_{}\frac{{\left(\frac{1}{\sigma \sqrt{2\pi }}e^{-\frac{{\left(z_{j}-\mu_{i}\right)}^{2}}{2\sigma^{2}}}\right)}^{1+\frac{2\kappa }{1+\kappa }}}{\int_{}{\left(\frac{1}{\sigma \sqrt{2\pi }}e^{-\frac{{\left(z_{j}-\mu_{i}\right)}^{2}}{2\sigma^{2}}}\right)}^{1+\frac{2\kappa }{1+\kappa }}dz_{j}}\cdot \left({\left(\frac{1}{\sigma \sqrt{2\pi }}e^{-\frac{{\left(z_{j}-\mu_{i}\right)}^{2}}{2\sigma^{2}}}\right)}^{-\frac{2\kappa }{1+\kappa }}-{\left(\frac{1}{\sqrt{2\pi }}e^{-\frac{{z_{j}}^{2}}{2}}\right)}^{-\frac{2\kappa }{1+\kappa }}\right)dz_{j} \end{array}$$

*is the generalized (coupled) KL-divergence in the original loss function in Equation (6), and*

(12) $$H_{\kappa }\left(x,y\right)\;\equiv -\frac{1}{2L}\sum_{l=1}^{L}\sum_{i=1}^{n_{x}}\left(x_{i}\ln_{\kappa }\left({\left(y_{i}\right)}^{\frac{2}{1+\kappa }}\right)\;+\;\left(1-x_{i}\right)\ln_{\kappa }\left({\left(1-y_{i}\right)}^{\frac{2}{1+\kappa }}\right)\right)$$

*is the generalized reconstruction loss (coupled cross-entropy) in the original loss function in Equation (6).*

In the next section, we show preliminary experimental evidence that the negative coupled ELBO can be used to improve the robustness and accuracy of the variational inference. We show that increasing the coupling parameter of the loss function has the effect of increasing the Accuracy (8) and Robustness (9) metrics of the generated data. Additionally, we show that the improvement in the generation process is not at the expense of the divergence between the posterior and the prior latent distributions. Thus, the overall ELBO is improved, indicating an improvement in the approximate variational inference. Furthermore, in Section 6, we show that improvements are more substantial when the algorithm is seeded by images from the corrupted MNIST database. While the experimental results of this report focus on a two-layer dense neural network and the (corrupted)-MNIST datasets, the generalization of information-theoretic cost functions for machine learning training is applicable to a broader range of architectures and datasets. For instance, the CIFAR-10 reconstruction is typically processed with a deep neural network [30] and is planned for future research.

## 4. Results Using the MNIST Handwritten Numerals

The MNIST handwritten digit database is a large database of handwritten digits consisting of a training set of 60,000 images and a test set of 10,000 images widely used for evaluating machine learning and pattern recognition methods. The digits have been size-normalized and centered in fixed-size images. Each image in the database contains 28 by 28 grayscale pixels. Pixel values vary from 0 to 255. Zero means the pixel is white, or background, while 255 means the pixel is black, or foreground [31]. In this and the next section, we examine the performance of the coupled VAE algorithm in reconstructing images of the MNIST database. In Section 6, we show the stability of the coupled VAE when reconstruction is distorted by samples from the corrupted MNIST database.

For this research, we used the MNIST database as the input since it was used in the traditional VAE. Specifically, input x is a batch of 28 by 28 pixel photos of handwritten numbers. The encoder encodes the data, which are 784-dimensional for each image in a batch into the latent layer space. For our experiment, the dimension of the latent variable **z** can be from 2 to 20. Taking the latent layers **z** as the input, the probability distribution of each pixel is computed using a Bernoulli or Gaussian distribution by the decoder. The decoder outputs the corresponding 784 parameters to reconstruct an image. We used specific numbers of images from the training set as the batch size and a fixed number of epochs. Additionally, for the learned MNIST manifold, visualizations of learned data and reproduced results were plotted. The algorithm and experiments were developed with Python and the TensorFlow library. Our Python code can be found in the Data Availability Statement.

**Figure 4:**
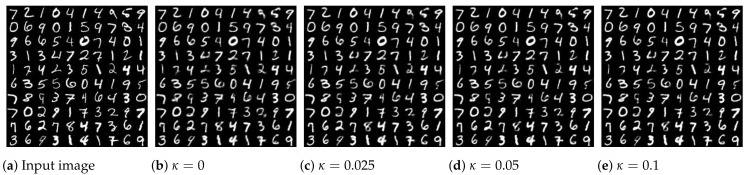
(**a**) The MNIST input images and (**b**) the output images generated by the original VAE. (**c**–**e**) The output images generated by the modified coupled VAE model show small improvements in detail and clarity. For instance, the fifth digit in the first row of the input images is ‘4’, but the output image in the original VAE is more like ‘9’ rather than ‘4’, while the coupled VAE method produced ‘4’ correctly.

The input images and output images for different values of coupling κ are shown in Figure 4. κ=0 represents the original VAE model. Compared with the original algorithm, output images generated by the modified coupled VAE model show small improvements in detail and clarity. For instance, the fifth digit in the first row of the input images is ‘4’, but the output image in the original VAE is more like ‘9’ rather than ‘4’, while the coupled VAE method generates ‘4’ correctly. For the seventh digit ‘4’ in the first row, the generated image in the coupled VAE has an improved clarity compared to the traditional VAE.

**Figure 5:**
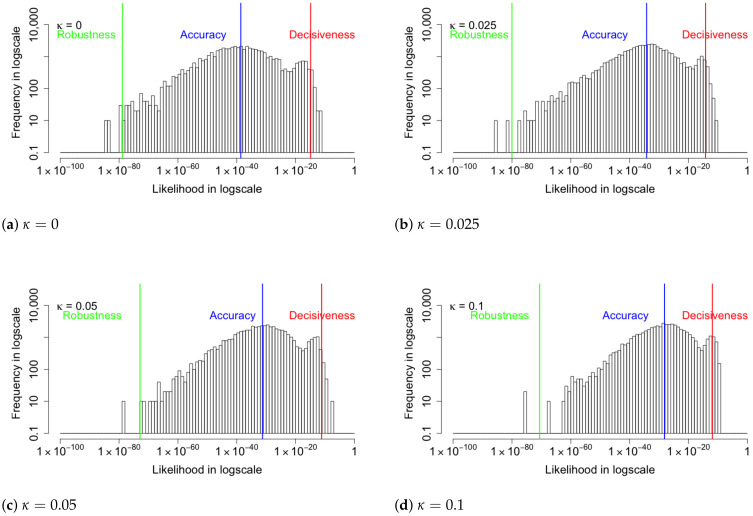
The histograms of likelihood for the reconstruction of the input images with various coupling κ values. The red, blue, and green lines represent the arithmetic mean (Decisiveness), geometric mean (Accuracy), and −2/3 mean (Robustness), respectively. The minimal value of the Robustness metric indicates that the original VAE suffers from poor robustness. As κ increases, the Robustness and Accuracy improve while the Decisiveness is mostly unchanged.

Figure 5 shows the likelihood histograms for 5000 input images with coupling values of κ=0,0.025,0.05,0.1. The red, blue, and green lines represent the arithmetic mean (decisiveness), geometric mean (central tendency), and −2/3 mean (robustness), respectively. When κ=0, the minimal value of the Robustness metric indicates that the original VAE suffers from poor robustness. As κ becomes large, the geometric mean and the −2/3 mean metrics start to increase while the arithmetic mean metric mostly stays the same. Since the probability of producing a correct image by a uniform random sampling is $\frac{1}{2^{28\times 28}}=9.8\times 10^{-237}$, the accuracy achieved by the VAE algorithm is significantly improved, even though the absolute value of the Accuracy metric seems small. As the coupling κ increases, the coupled loss function approaches infinity faster. This eventually causes computational errors. For instance, when κ=0.2, the loss function has a computational error at the 53^rd^ epoch; when κ=0.5, the loss function has a computational error at the 8^th^ epoch. Further investigations of the computational bounds of the algorithm are planned. The specific relationship between coupling κ and probabilities for input images is shown in Table 1. The increased Robustness metric shows that the modified loss does improve the robustness of the the reconstructed image. In the next section, we also examine the performance of the divergence between the posterior and prior distributions of the latent layer.

Furthermore, compared with the original VAE model, the geometric mean, which measures the accuracy of the input image likelihood, is larger for the coupled algorithm. The improvement of this metric means that the input images (truth) are assigned to higher likelihoods on average by the coupled VAE model.

**Figure 6:**
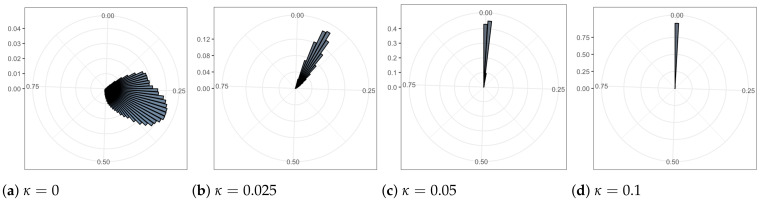
The rose plots of the various standard deviation values in 20 dimensions. The range and average values of these standard deviations are reduced as coupling increases.

The standard deviation σ of latent variables z is shown in rose plots in Figure 6. The angular position of a bar represents the value of σ, clockwise from 0 to 1. The radius of the bar measures the frequency of different σ values from 0 to 100. As the coupling κ increases, the range and the average value of these standard deviations decrease. To be specific, when κ=0, σ of all dimensions in all 5000 batches ranges from 0.09 to 0.72; when κ=0.025, σ ranges from 0.02 to 0.3; when κ=0.05, σ ranges from 0.001 to 0.09; when κ=0.1, σ ranges from 0.00007 to 0.06.

We note that as coupling parameter κ increases, the variability of the latent space diminishes. One possible method to address this problem is to use heavy-tail distribution in the latent layer. Chen et al. [32] and Nelson [23] used the Student’s *t* as the distribution [33] of the latent layer to incorporate heavy-tail decay.

**Figure 7:**
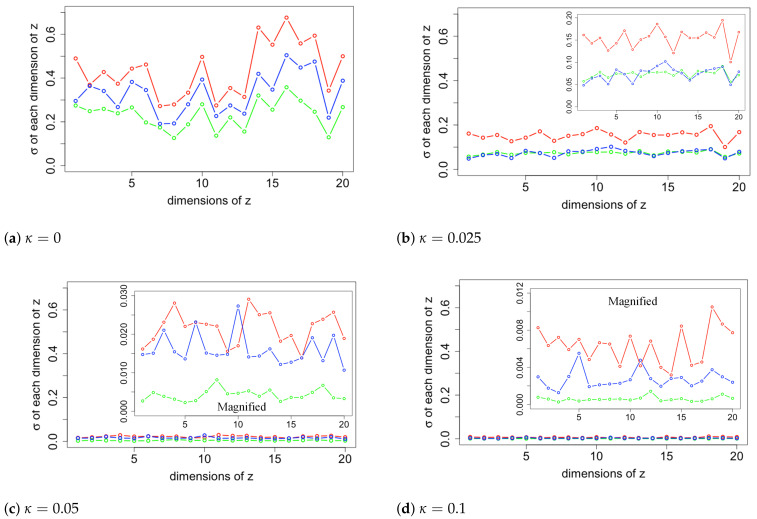
The standard deviation of latent variable samples near the three generalized mean metrics. The red, blue, and green lines represent samples near the Decisiveness, Accuracy, and Robustness, respectively. As κ increases, values of σ fluctuate less and decrease toward 0. Magnified plots are shown to visualize the results further.

**Figure 8:**
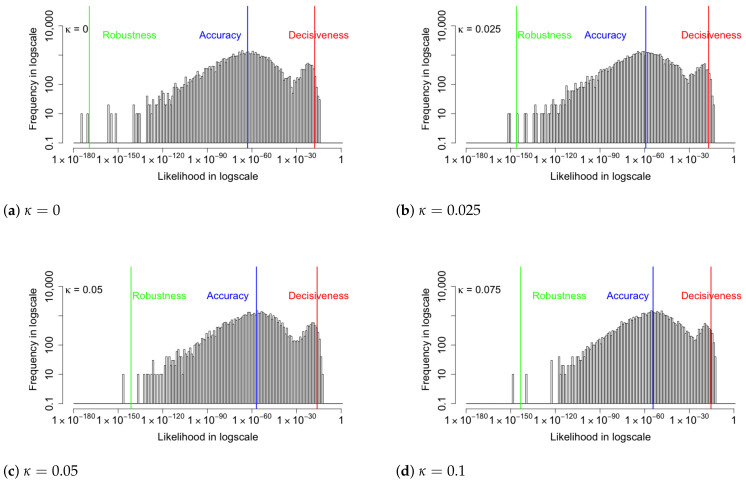
The histogram likelihood plots with a two-dimensional latent variable. Like the 20-D model, the increased values of the arithmetic mean metric and −2/3 mean metric show that the accuracy and robustness of the VAE model have been improved.

We choose samples in which the likelihoods of input images are close to the three metrics and plot the standard deviation σ of each dimension of the latent variable z of these samples in Figure 7. The red, blue, and green lines represent samples near the decisiveness, accuracy, and robustness, respectively. It shows that when κ=0, the standard deviations of z range from 0.1 to 0.7. However, as κ increases, values of σ fluctuate less and decrease toward 0. Magnified plots are shown to visualize the results further. The general trend for σ is to be more significant for samples near decisiveness, intermediate near the accuracy, and smaller for samples near robustness. An exception is κ=0.025, where σ overlaps for samples near the robustness and accuracy. The histogram likelihood plots with a two-dimensional latent variable are shown in Figure 8. The increased values of the arithmetic mean metric and −2/3 mean metric show that the accuracy and robustness of the output MNIST images in the VAE model have been improved, consistent with the result in the 20-D model. While the performance improvements are modest, we will show in Section 6 that the performance improvements when the algorithm is seeded with corrupted images is much more substantial. First, we provide a visualization of the changes in the latent distribution using two dimensions.

## 5. Visualization of Latent Distribution

In order to understand the relationship between increasing coupling of the loss function with the means and the standard deviations of the Gaussian model, we examine a two-dimensional model which can be visualized. Compared with the high-dimensional model, the probability likelihoods for the two-dimensional model are lower, indicating that the higher dimensions do improve the model. Nevertheless, like the 20-dimensional model, the distribution of likelihood is compressed toward higher values as the coupling increases and, therefore, can be used to analyze the results further. Larger likelihood of input images along with both means closer to the origin and smaller standard deviations of latent variables are the primary characteristics as the coupling parameter of the loss function is increased. As a result, both the robustness and accuracy of likelihoods increase. To be specific, when κ increases from 0 to 0.075, the geometric mean metric increases from $1.20\times 10^{-63}$ to $4.67\times 10^{-55}$, and the −2/3 mean metric increases from $5.03\times 10^{-170}$ to $5.17\times 10^{-144}$, while the arithmetic metric does not change very much. In this case, the reconstructed images have a higher probability of replicating the input image using the coupled VAE method.

**Figure 9:**
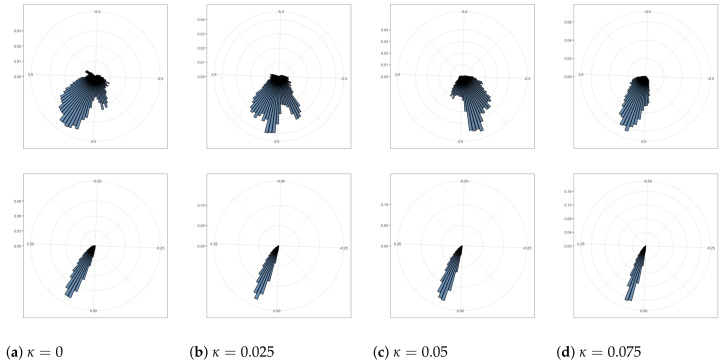
The rose plots of the various mean (above four figures) and standard deviation (below four figures) values in 2 dimensions. The range of means is reduced and mean values become closer to 0 as coupling increases.

The rose plots in Figure 9 show that the range and variability of the mean values of latent variables decrease as the coupling κ increases. From the view of means, the posterior distribution of the latent space is closer to the prior, the standard Gaussian distribution. From the view of standard deviations, the posterior distribution of the latent space is further from the prior.

**Figure 10:**
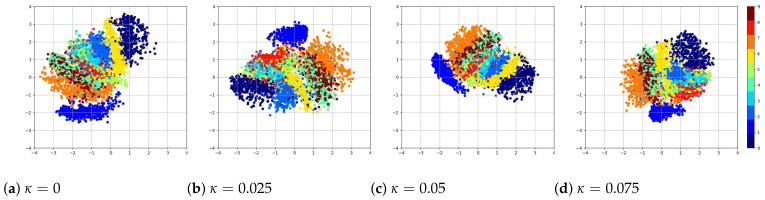
The plot of the latent space of VAE trained for 200 epochs on MNIST with various κ values. Different numerals cluster together more tightly as coupling κ increases.

**Figure 11:**
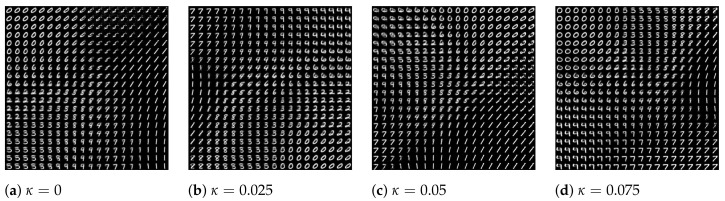
The plot of visualization of learned data manifold for generative models with the axes as the values of each dimension of latent variables. The distinct digits each exist in different regions of the latent space and smoothly transform from one digit to another.

The latent space plots shown in Figure 10 are the visualizations of images of the numerals from 0 to 9. Images are embedded in a 2D map where the axis is the values of the 2D latent variable. The same color represents images that belong to the same numeral, and they cluster together since they have higher similarity to each other. The distances between spots represent the similarities of images. The latent space plots show that the different clusters shrink together more tightly when coupling becomes larger. The plots shown in Figure 11 are the visualizations of the learned data manifold generated by the decoder network of the coupled VAE model. A grid of values from a two-dimensional Gaussian distribution is sampled. The distinct digits each exist in different regions of the latent space and smoothly transform from one digit to another. This smooth transformation can be quite useful when the interpolation between two observations is needed. Additionally, the distribution of distinct digits in the plot becomes more even, and the sharpness of the digits increases when κ increases.

As shown in Table 2, as the coupling increases from 0 to 0.075, the negative ELBO (the loss) decreases from 172.3 to 146.7, the coupled KL-divergence decreases from 5.8 to 5.6, and the coupled reconstruction loss decreases from 166.5 to 141.1. It shows that the reconstruction loss plays a dominant role (with proportion over 96%), while the divergence term has a much lower effect (with proportion under 4%) in the loss function. The overall improvement of coupled loss is based on both the smaller coupled KL-divergence and the smaller coupled reconstruction error, instead of a trade-off between them. There is a high degree of variability in this improvement, so there are reasons to be cautious about the degree of improvement. In addition, since the coupled loss function is adjusting the metric, the property being measured is also adjusting. Part of our future research plan is to explore how the relative performance between the reconstruction and the latent space can be compared.

## 6. Performance with Corrupted Images

**Figure 12:**
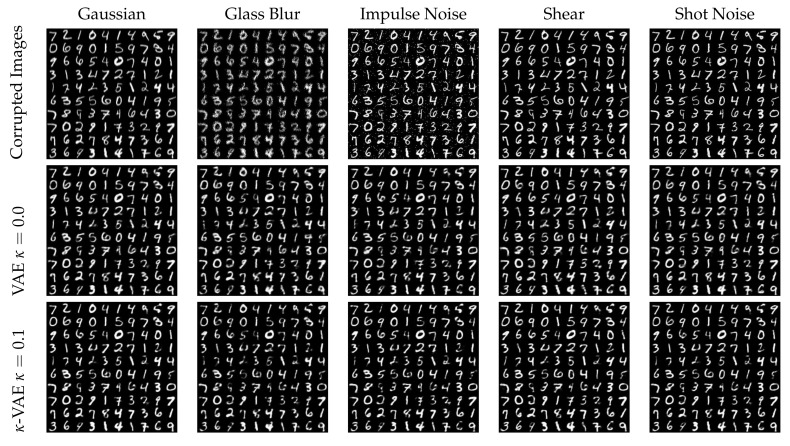
The images with 5 different corruptions are shown in the first row. The reconstructed images when κ=0.0 and κ=0.1 are shown in the second and third rows, respectively. The qualitative visual improvement in clarity using the coupling is modest.

We also evaluate the performance of the coupled VAE algorithm when keyed by images from the corrupted MNIST (C-MNIST) dataset [34]. The reconstructed images under 5 different corruptions: Gaussian corruption, glass blur corruption, impulse noise corruption, shot noise corruption and shear corruption, with two coupling values κ=0.0 and κ=0.1 are shown in the Figure 12. Based on the visualization of the generated images, the qualitative visual improvement in clarity using the coupling is modest.

**Figure 13:**
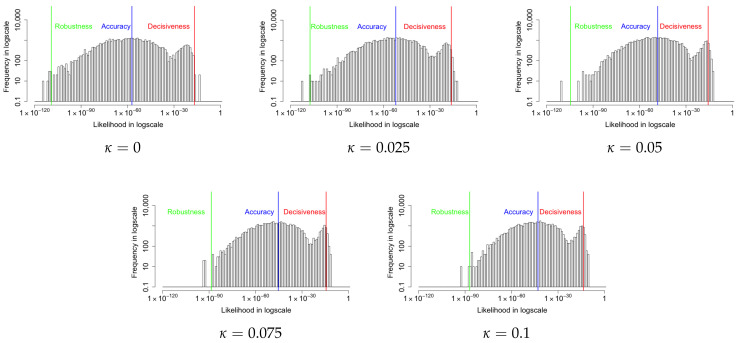
The histograms of marginal likelihood for the MNIST images with **Gaussian** corruption shown. All three metrics increase as the coupling parameter κ increases. The robustness improves the most, central tendency is the next, and decisiveness has the least improvement. From κ=0.0 to κ=0.1, the Robustness improves from $10^{-109.2}$ to $10^{-87.0}$, the Accuracy improves from $10^{-57.2}$ to $10^{-42.9}$, and the Decisiveness improves from $10^{-16.8}$ to $10^{-13.6}$.

We also conduct the further analyses for the performance of the coupled VAE with each corruption. For the MNIST images with Gaussian corruption, as shown in the Figure 13, when the coupling parameter κ increases, all the three metrics—robustness, central tendency, and decisiveness—increase. The robustness improves the most, central tendency is the next, and decisiveness has the least improvement. Furthermore, we confirm that the reconstruction improvement is not a trade-off with latent distribution divergence, as shown in Table 3. This is in contrast to the β-VAE [11] method which merely alters the weight between the reconstruction and divergence components of the negative ELBO cost function.

In the Table 3, analyses of the components of the coupled ELBO are provided. Comparisons as the coupling changes are somewhat confusing because the metric itself is changing. Therefore, as the coupling increases the measure of performance is more difficult. Nevertheless, there is still an overall tendency towards improved performance, even with this caveat. The second column shows that the coupled KL-divergence initially increases when moving away from the standard VAE design with κ=0, however, it then steadily decreases with increasing κ. This may be due to the distinct difference between the logarithm and even a slight deviation from the logarithm. The coupled reconstruction loss (column three) shows steady improvement. The overall negative coupled ELBO shows consistent improvement as the coupling increases. The relative importance of the divergence and reconstruction varies as the coupling increases but in each case it is approximately a 15% to 85% relative weighting.

**Figure 14:**
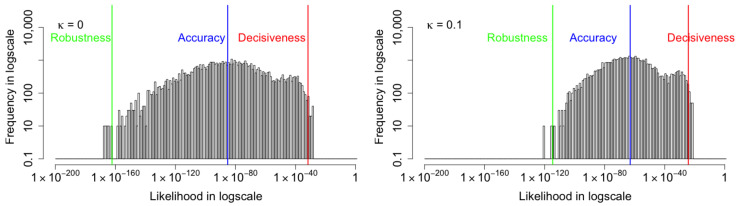
The histograms of marginal likelihood for the MNIST images with **glass blur** corruption are shown. All the three metrics increase as the coupling parameter κ increases from 0 to 0.1.

**Figure 15:**
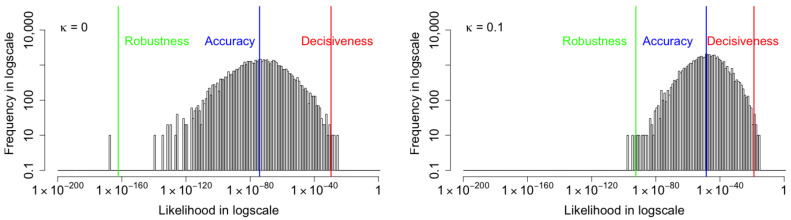
The histograms of marginal likelihood for the MNIST images with **impulse noise** corruption are shown. All the three metrics increase as the coupling κ increases from 0 to 0.1.

**Figure 16:**
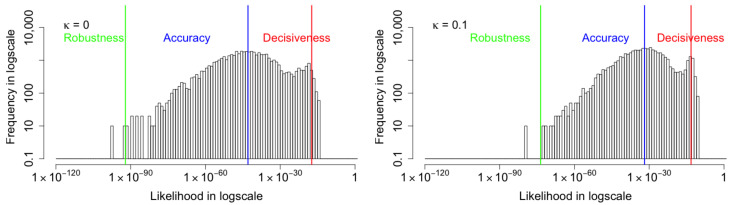
The histograms of marginal likelihood for the MNIST images with **shot noise** corruption are shown. All the three metrics increase as the coupling parameter κ increases from 0 to 0.1.

**Figure 17:**
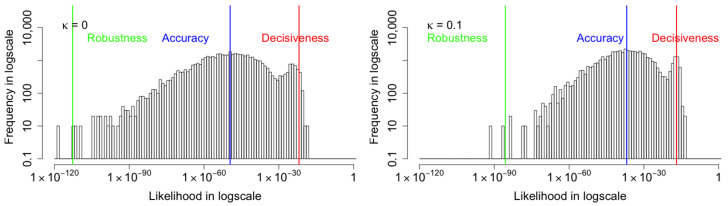
The histograms of marginal likelihood for the MNIST images with **shear** corruption are shown. All the three metrics increase as the coupling parameter κ increases from 0 to 0.1.

The improvement of the three metrics with glass blur corruption, impulse noise corruption, shot noise corruption and shear corruption is also observed and shown in Figure 14, Figure 15, Figure 16 and Figure 17, respectively. Similar to the Gaussian corruption, all the three metrics gradually increase as the coupling parameter κ increases from 0 to 0.1. The respective analyses of the components of the coupled ELBO with glass blur corruption, impulse noise corruption, shot noise corruption and shear corruption are provided in Table 4, Table 5, Table 6 and Table 7. The four corruptions share the consistent results, the coupled KL-divergence initially increases when moving away from the standard VAE design with κ≤0.025, but it then steadily decreases with increasing κ. The overall negative coupled ELBO shows consistent improvement as κ increases. It means that if the coupling parameter is relatively large (>0.025), both the KL-divergence and the reconstruction loss will be improved, thus the overall improvement of the algorithm is not a trade-off between the reconstruction accuracy and the latent distribution divergence.

## 7. Discussion and Conclusions

This investigation sought to determine whether the accuracy and robustness of variational autoencoders can be improved using certain statistical methods developed within the area of complex systems theory. Our investigation provides evidence that the tail shape of the negative evidence lower bound can be controlled in such a way that the cost of outlier events is adjustable. We refer to this method as a coupled VAE, since the control parameter models the nonlinear deviation from the exponential and logarithmic functions of linear analysis. A positive coupling parameter increases the cost of these tail events and thereby trains the algorithm to be robust against such outliers. Additionally, this improves both the accuracy of reconstructed images and reduces the divergence of the posterior latent distribution from the prior. We have been able to document this improvement using the histogram of the reconstructed marginal likelihoods. Metrics of the histogram are formed from the arithmetic mean, geometric mean, and −2/3 mean, which represent Decisiveness, Accuracy, and Robustness, respectively. Both the accuracy and the robustness are improved by increasing the coupling of the loss function. There is a limit to such increases in the coupling beyond which the training process no longer converges.

These performance improvements have been evaluated for the MNIST handwritten numeral dataset and its corrupted modification C-MNIST. We used a two-layer dense neural network for the encoder/decoder. The latent layer is a 20-dimensional Gaussian distribution and for visualization a 2-dimensional distribution was also examined. Without the corruption, we observed improvements in both components of the negative coupled ELBO loss function, namely the image reconstruction loss (marginal likelihood) and the latent distribution (divergence between the prior and posterior). Thus, the coupled VAE is able to improve the model representation, rather than just trading off reconstruction and divergence performance, as does the highly cited β-VAE design. The likelihood of the reconstructed image matching the original improves in Accuracy by $10^{10}$ and in Robustness by $10^{8}$ when the coupling parameter was increased from κ=0 (the standard VAE) to κ=0.1 (the largest value of the coupled VAE reported). The Decisiveness did not change significantly, though there is potential that negative values of the coupling could influence this metric. The performance improvements when the algorithm is seeded by the C-MNIST dataset are far more significant, demonstrating the improved stability of the algorithm. All five corruptions examined (Gaussian, glass blur, impulse noise, shot noise, and shear) show significant improvement in Robustness and Accuracy and some improvement in the Decisiveness. For example, under the Gaussian corruption, the improvements in the reconstruction likelihood for Accuracy are $10^{14}$ and those for the Robustness are $10^{20}$ when the coupling parameter is increased from κ=0 (the standard VAE) to κ=0.1. The significant improvement in Robustness using the corrupted MNIST dataset demonstrates that the coupled negative ELBO cost function reduces the risk of overfitting by forcing the network to learn general solutions that are less likely to create outliers.

The modifications of the latent posterior distributions have been further examined using a two-dimensional representation. We show that the latent variables have both a tighter distribution of the mean about its prior value of zero, and a movement of standard deviations towards zero, away from the prior of one, as coupling κ increases. Overall, the coupled KL-divergence does indeed decrease as the coupling is increased, indicating improvement in the latent representation. Thus, improvements in the reconstruction evident from both visual clarity of images and increased accuracy in measured likelihoods are not due to a trade-off with the latent representation. Rather, the negative coupled ELBO metric shows improvement in both latent layer divergence and output image reconstruction. This improvement in the two components of the evidence lower bound provides evidence that the coupled VAE improves the approximate variational inference of the model.

In future research, we plan to study the coupled Gaussian distribution as the prior and posterior distribution of the latent layer. This may be helpful for achieving greater separation between the images into distinct clusters similar to what has been achieved with t-stochastic neighborhood embedding methods [35]. If so, it may be possible to improve the decisiveness of the likelihoods in addition to further improvements in the accuracy and robustness. Since our approach generalizes the training of the decoder and encoder networks, it is expected to be seamlessly applicable to other datasets and neural network architectures. We are conducting research to apply our method to a convolutional neural network design that can process more complex datasets such as CIFAR-10. This first demonstration of the coupled ELBO cost function has provided experimental results applied to a shallow neural network but the approach is also applicable to the training of deep neural networks.

## Figures and Tables

**Table 1:** The Decisiveness, Accuracy, and Robustness of the reconstruction likelihood as a function of the coupling κ.

Coupling κ	Decisiveness	Accuracy	Robustness
0	$1.31\times 10^{-15}$	$2.41\times 10^{-39}$	$1.40\times 10^{-79}$
0.025	$6.61\times 10^{-15}$	$5.89\times 10^{-35}$	$9.91\times 10^{-81}$
0.05	$7.18\times 10^{-12}$	$5.80\times 10^{-32}$	$1.31\times 10^{-73}$
0.1	$1.34\times 10^{-12}$	$7.09\times 10^{-29}$	$2.57\times 10^{-71}$

**Table 2:** Components of coupled ELBO with a 2-dimensional latent layer under different values of coupling. The improvement in the coupled KL-divergence is very slight, while it is larger for the coupled reconstruction loss.

Coupling κ	Coupled KL-Divergence	Coupled RE Loss	Coupled ELBO	KL Proportion	RE Proportion
0	5.8 +/− 1.7	166.5 +/− 52.2	172.3	3.38%	96.62%
0.025	5.7 +/− 1.6	156.4 +/− 49.8	162.1	3.53%	96.47%
0.05	5.6 +/− 1.6	149.2 +/− 46.6	154.8	3.61%	96.39%
0.075	5.6 +/− 1.7	141.1 +/− 44.6	146.7	3.82%	96.18%

**Table 3:** The components of the coupled ELBO under **Gaussian** corruptions are provided in the table. The coupled KL-divergence initially increases when moving away from the standard VAE design with κ=0 to κ=0.025, however, it then steadily decreases with increasing κ. The coupled reconstruction loss (column three) shows steady improvement. The overall negative coupled ELBO shows consistent improvement as the coupling increases. The relative importance of the divergence and reconstruction varies as the coupling increases but in each case it is approximately a 15% to 85% relative weighting.

Coupling κ	Coupled KL-Divergence	Coupled RE Loss	Coupled ELBO	KL Proportion	RE Proportion
κ=0	23.9 +/− 3.8	131.6 +/− 40.7	155.5	15.34%	84.66%
κ=0.025	29.6 +/− 2.3	119.9 +/− 38.5	149.5	19.80%	80.20%
κ=0.05	26.0 +/− 0.9	111.1 +/− 36.5	137.1	18.94%	80.06%
κ=0.075	21.4 +/− 0.5	104.4 +/− 34.3	125.8	16.98%	83.02%
κ=0.1	18.4 +/− 0.6	98.9 +/− 32.7	117.3	15.71%	84.28%

**Table 4:** The components of the coupled ELBO under **glass blur** corruptions are provided in the table. The coupled KL-divergence initially increases when moving away from the standard VAE design with κ≤0.025, but it then steadily decreases with increasing κ. The coupled reconstruction loss shows steady improvement. The overall negative coupled ELBO shows consistent improvement as κ increases.

Coupling κ	Coupled KL-Divergence	Coupled RE Loss	Coupled ELBO	KL Proportion	RE Proportion
κ=0	22.3 +/− 3.5	196.1 +/− 55.3	218.4	10.19%	89.81%
κ=0.025	29.4 +/− 2.0	178.8 +/− 50.1	208.2	14.12%	85.88%
κ=0.05	25.5 +/− 0.7	164.1 +/− 45.7	189.6	13.44%	86.56%
κ=0.075	20.9 +/− 0.4	154.0 +/− 43.0	174.9	11.96%	88.04%
κ=0.1	18.0 +/− 0.4	145.1 +/− 40.0	163.1	11.05%	88.95%

**Table 5:** The components of the coupled ELBO under **impulse noise** corruptions are provided in the table. The coupled KL-divergence initially increases when moving away from the standard VAE design with κ≤0.025, but it then steadily decreases with increasing κ. The overall negative coupled ELBO shows consistent improvement as κ increases.

Coupling κ	Coupled KL-Divergence	Coupled RE Loss	Coupled ELBO	KL Proportion	RE Proportion
κ=0	24.2 +/− 3.8	170.7 +/− 34.7	195.0	12.43%	87.57%
κ=0.025	29.9 +/− 2.2	148.0 +/− 31.0	177.9	16.81%	83.19%
κ=0.05	26.0 +/− 0.8	131.6 +/− 28.5	157.7	16.52%	83.48%
κ=0.075	21.4 +/− 0.6	120.9 +/− 26.7	142.3	15.05%	84.95%
κ=0.1	18.5 +/− 0.6	111.8 +/− 25.2	130.3	14.21%	85.79%

**Table 6:** The components of the coupled ELBO under **shot noise** corruptions are provided in the table. The coupled KL-divergence increases when moving away from the standard VAE design with κ≤0.025, but it then steadily decreases with increasing κ. The coupled reconstruction loss shows steady improvement. The overall negative coupled ELBO shows consistent improvement as κ increases.

Coupling κ	Coupled KL-Divergence	Coupled RE Loss	Coupled ELBO	KL Proportion	RE Proportion
κ=0	23.9 +/− 3.8	98.9 +/− 28.3	122.8	19.45%	80.55%
κ=0.025	29.9 +/− 2.4	88.9 +/− 26.2	118.8	25.14%	74.86%
κ=0.05	26.1 +/− 1.0	81.8 +/− 25.0	108.0	24.21%	75.80%
κ=0.075	21.6 +/− 0.7	77.6 +/− 23.9	99.2	21.80%	78.20%
κ=0.1	18.6 +/− 0.6	73.4 +/− 22.8	92.0	20.17%	79.83%

**Table 7:** The components of the coupled ELBO under **shear** corruptions are provided. The coupled KL-divergence increases when moving away from the standard VAE design with κ≤0.025, but it then steadily decreases with increasing κ. The coupled reconstruction loss shows steady improvement. The overall negative coupled ELBO shows consistent improvement as κ increases.

Coupling κ	Coupled KL-Divergence	Coupled RE Loss	Coupled ELBO	KL Proportion	RE Proportion
κ=0	24.8 +/− 4.0	114.1 +/− 31.7	138.9	17.85%	82.15%
κ=0.025	30.4 +/− 2.4	102.3 +/− 29.0	132.7	22.92%	77.08%
κ=0.05	26.1 +/− 0.9	94.7 +/− 27.5	120.8	21.61%	78.39%
κ=0.075	21.8 +/− 0.7	89.5 +/− 26.3	111.3	19.61%	80.39%
κ=0.1	18.6 +/− 0.6	84.9 +/− 24.9	103.5	17.97%	82.03%

## Data Availability

The algorithm and data can be accessed at https://github.com/Photrek/Coupled-VAE-Improved-Robustness-and-Accuracy-of-a-Variational-Autoencoder, accessed on 5 February 2022.

## Appendix A

### Appendix A.1. Derivation of Negative Coupled ELBO

Generalizing the negative ELBO is accomplished using the principles of nonlinear statistical coupling (NSC) to generalize information theory. As described in Section 2.1, the negative ELBO consists of two components, the KL-divergence between the prior and posterior latent distribution, and the cross-entropy or negative log-likelihood of the reconstructed image in relation to the original image. NSC is an approach to modeling the statistics of complex systems that unifies heavy-tailed distributions, generalized information metrics, and fusion of information. Its application to the cost functions of a VAE provides control over the trade-off between decisive and robust generative models. Decisive refers to the characteristic of confident probabilities and robust refers to the characteristic of dampening extremes in the probabilities.

In the VAE algorithm, the loss function consists of the KL-divergence between the posterior approximation $q\left(z|x^{\left(i\right)}\right)$ and a prior $p\left(z\right)$ and the cross-entropy between the reported probabilities and the training sample distribution.

(A1) $$L\left(x^{\left(i\right)}\right)=D_{KL}\left(q\left(z|x^{\left(i\right)}\right)\|\;p\left(z\right)\right)\;-\;\frac{1}{L}\sum_{l=1}^{L}\left(\log p\left(x^{\left(i\right)}|z^{\left(i,l\right)}\right)\right),$$

where *L* is the number of reconstructions per test sample, and the KL-divergence is given by

(A2) $$D_{KL}\left(q\left(z|x^{\left(i\right)}\right)\|\;p\left(z\right)\right)\;=\int_{}q\left(z|x^{\left(i\right)}\right)\left(\log q\left(z|x^{\left(i\right)}\right)-\log p(z)\right)dz.$$

Even though $x^{\left(i\right)}$ given z is a grayscaled value, which is not Bernoulli distributed, we can still use the probability mass function of Bernoulli distribution, then the cross entropy term is given by

(A3) $$-\frac{1}{L}\sum_{l=1}^{L}\left(\log p\left(x^{\left(i\right)}|z^{\left(i,l\right)}\right)\right)\;=-\frac{1}{L}\sum_{l=1}^{L}\sum_{i=1}^{n_{x}}\left[x_{i}\log y_{i}+\left(1-x_{i}\right)\log\left(1-y_{i}\right)\right],$$

where $y=\mathrm{Sigmod}(f_{2}\left(\tanh\left(f_{1}\left(z\right)\right)\right))$ while $f_{1}$ and $f_{2}$ are linear models and $n_{x}$ is the dimensionality of x.

The negative ELBO loss function is modified by coupled generalizations of the KL-divergence and cross-entropy. The purpose is to increase the weighting of rare events in the training dataset and thereby improve the robustness of the VAE model. The connection with the assessment metrics defined in Section 3.1 is that the power of the generalized mean can be decomposed into functions of the coupling and second parameter α, related to the power in the distribution of the random variable. For Gaussians and their generalizations, known as coupled Gaussians, α=2. Making use of $r\left(\kappa ,\alpha ,d\right)=\frac{-\alpha \kappa }{1+d\kappa }$ with α=2, the generalized mean is ${\left(\sum p_{i}^{1+r}\right)}^{\frac{1}{r}}={\left(\sum p_{i}^{1-\frac{2\kappa }{1+\kappa }}\right)}^{-\frac{1+\kappa }{2\kappa }}$. When the coupling κ→0, the generalized mean is asymptotically equal to the geometric mean.

The coupled entropy function takes the form of a generalized logarithmic function applied to the generalized mean [22].

(A4) $$H_{\kappa }\left(p\right)\equiv \frac{1}{2}\ln_{\kappa }\left({\left(\sum p_{i}^{1+\frac{2\kappa }{1+\kappa }}\right)}^{\frac{-1}{\kappa }}\right)\;\equiv \frac{p_{i}^{1+\frac{2\kappa }{1+\kappa }}}{2\sum p_{i}^{\frac{1+2\kappa }{1+\kappa }}}\ln_{\kappa }p_{i}^{-\frac{2}{1+\kappa }}\equiv \frac{1}{2\kappa }\left({\left(\sum p_{i}^{\frac{1+3\kappa }{1+\kappa }}\right)}^{-1}-1\right),$$

where $\ln_{\kappa }\left(x\right)$ is the generalization of the logarithm function in Equation (A15).

Similar to the generalization of coupled entropy function, the generalized logarithmic is applied to the KL-divergence. The first term in KL-divergence becomes

(A5) $$-\int_{}q(z|x^{\left(i\right)})\log q(z|x^{\left(i\right)})dz\Rightarrow \frac{1}{2}\prod_{j=1}^{n_{z}}\int_{}\frac{{q(z_{j}|x^{\left(i\right)})}^{1+\frac{2\kappa }{1+\kappa }}}{\int_{}q(z_{j}|x^{\left(i\right)}){}^{1+\frac{2\kappa }{1+\kappa }}dz_{j}}\ln_{\kappa }\left(q(z_{j}|x^{\left(i\right)}){}^{-\frac{2}{1+\kappa }}\right)dz_{j},$$

and the second term in KL-divergence becomes

(A6) $$-\int_{}q(z|x^{\left(i\right)})\log p(z)dz\Rightarrow \frac{1}{2}\prod_{j=1}^{n_{z}}\int_{}\frac{{q(z_{j}|x^{\left(i\right)})}^{1+\frac{2\kappa }{1+\kappa }}}{\int_{}q(z_{j}|x^{\left(i\right)}){}^{1+\frac{2\kappa }{1+\kappa }}dz_{j}}\ln_{\kappa }\left(p(z_{j}){}^{-\frac{2}{1+\kappa }}\right)dz_{j},$$

Therefore, the coupled divergence with $n_{z}$ as the dimensionality of z can be written as

(A7) $$\begin{array}{cc} \; & D_{\kappa }\left(q\left(z|x^{\left(i\right)}\right)\|\;p\left(z\right)\right)\\ \equiv & \prod_{j=1}^{n_{z}}\int_{}\frac{{q(z_{j}|x^{\left(i\right)})}^{1+\frac{2\kappa }{1+\kappa }}}{\int_{}q(z_{j}|x^{\left(i\right)}){}^{1+\frac{2\kappa }{1+\kappa }}dz_{j}}\frac{1}{2}\left(\ln_{\kappa }\left(q{\left(z_{j}|x^{\left(i\right)}\right)}^{-\frac{2}{1+\kappa }}\right)-\ln_{\kappa }\left(p{\left(z_{j}\right)}^{-\frac{2}{1+\kappa }}\right)\right)dz_{j}\\ = & \prod_{j=1}^{n_{z}}\frac{1}{2\kappa }\int_{}\frac{{\left(\frac{1}{\sigma \sqrt{2\pi }}e^{-\frac{{\left(z_{j}-\mu_{i}\right)}^{2}}{2\sigma^{2}}}\right)}^{1+\frac{2\kappa }{1+\kappa }}}{\int_{}{\left(\frac{1}{\sigma \sqrt{2\pi }}e^{-\frac{{\left(z_{j}-\mu_{i}\right)}^{2}}{2\sigma^{2}}}\right)}^{1+\frac{2\kappa }{1+\kappa }}dz_{j}}\cdot \left({\left(\frac{1}{\sigma \sqrt{2\pi }}e^{-\frac{{\left(z_{j}-\mu_{i}\right)}^{2}}{2\sigma^{2}}}\right)}^{-\frac{2\kappa }{1+\kappa }}-{\left(\frac{1}{\sqrt{2\pi }}e^{-\frac{{z_{j}}^{2}}{2}}\right)}^{-\frac{2\kappa }{1+\kappa }}\right)dz_{j} \end{array}.$$

The original cross-entropy can also be modified in a similar way. Applying the generalization of the logarithmic function, the terms $\log(y_{i})$ and $\log(1-y_{i})$ are modified to $\frac{1}{2}\ln_{\kappa }\left({\left(y_{i}\right)}^{\frac{2}{1+\kappa }}\right)$ and $\frac{1}{2}\ln_{\kappa }\left({\left(1-y_{i}\right)}^{\frac{2}{1+\kappa }}\right)$, thus

(A8) $$\log p\left(x^{\left(i\right)}|z^{\left(i,l\right)}\right)\;\Rightarrow \sum_{i=1}^{n_{x}}\left(x_{i}\frac{1}{2}\ln_{\kappa }\left({\left(y_{i}\right)}^{\frac{2}{1+\kappa }}\right)\;+\;\left(1-x_{i}\right)\frac{1}{2}\ln_{\kappa }\left({\left(1-y_{i}\right)}^{\frac{2}{1+\kappa }}\right)\right).$$

Therefore, the coupled cross-entropy is the generalization of the cross-entropy term in Equation (A14), which is defined as

(A9) $$H_{\kappa }\left(x_{i},y_{i}\right)\;\equiv -\frac{1}{2L}\sum_{l=1}^{L}\sum_{i=1}^{n_{x}}\left(x_{i}\ln_{\kappa }\left({\left(y_{i}\right)}^{\frac{2}{1+\kappa }}\right)\;+\;\left(1-x_{i}\right)\ln_{\kappa }\left({\left(1-y_{i}\right)}^{\frac{2}{1+\kappa }}\right)\right).$$

Adding Equations (A7) and (A9) gives the negative coupled ELBO,

(A10) $$L_{\kappa }\left(x^{\left(i\right)}\right)\;=D_{\kappa }\left(q\left(z|x^{\left(i\right)}\right)\|\;p\left(z\right)\right)+H_{\kappa }\left(x,y\right),$$

as defined in Equations (10)–(12).

### Appendix A.2. Origin of the Generalized Probability Metrics

The generalized probability metrics derive from a translation of a generalized entropy function back to the probability domain. Use of the geometric mean for Accuracy derives from the Boltzmann–Gibbs–Shannon entropy, which measures the average uncertainty of a system and is equal to the arithmetic average of the negative logarithm of the probability distribution,

(A11) $$H\left(P\right)\;\equiv -\sum_{i=1}^{N}p_{i}\ln p_{i}=-\ln\left(\prod_{i=1}^{N}p_{i}^{p_{i}}\right).$$

Translating the entropy back to the probability domain via the inverse of the negative logarithm, which is the exponential of the negative, results in the weighted geometric mean of the probabilities

(A12) $$P_{avg}\equiv \exp\left(-H\left(P\right)\right)\;=\exp\left(\ln\left(\prod_{i=1}^{N}p_{i}^{p_{i}}\right)\right)=\prod_{i=1}^{N}p_{i}^{p_{i}}.$$

The role of this function in defining the central tendency of the y-axis of a density is illustrated with the Gaussian distribution. Utilizing the continuous definition of entropy for a density $f\left(x\right)$ for a random variable *x*, the neutral accuracy or central tendency of the density is

(A13) $$f_{avg}\equiv \exp\left(-H\left(f\left(x\right)\right)\right)=\exp\left(\int_{X}f\left(x\right)\ln f\left(x\right)dx\right).$$

For the Gaussian, the average density is equal to the density at the mean plus the standard deviation $f\left(\mu \pm \sigma \right)$.

The use of the geometric mean as a metric for the neutral accuracy in the previous section is related to the cross-entropy between the reported probability of the algorithm and the probability distribution of the test set. The cross-entropy between a ‘quoted’ or predicted probability distribution q and the distribution of the test set p is

(A14) $$H\left(p,q\right)\;\equiv -\sum_{i}p_{i}\ln q_{i}.$$

In evaluating an algorithm, the actual distribution is defined by the test samples which, for equally probable independent samples, each have a probability of $p_{i}=\frac{1}{N}$. Translated to the probability domain, the cross-entropy becomes the geometric mean of the reported probabilities (8), thus showing that use of the geometric mean of the probabilities as a measure of Accuracy for reported probabilities is equivalent to the use of cross-entropy as a metric of forecasting performance.

Likewise, the use of the generalized mean as a metric for Robustness and Decisiveness derives from a generalization of the cross-entropy. While there are a variety of proposed generalizations to information theory, in [22,36,37,38], the Renyi and Tsallis entropies were both shown to translate to a generalized mean upon transformation to the probability domain. Here, we show that the derivation of this transformation uses the coupled entropy, which derives from the Tsallis entropy, but utilizes a modified normalization. The nonlinear statistical coupling (or simply the coupling) has been shown to (a) quantify the relative variance of a superstatistics model in which the variance of exponential distribution fluctuates according to a gamma distribution, and (b) be equal to the inverse of the degree of freedom of the Student’s *t* distribution. The coupling is related to the risk bias by the expression $r=\frac{-2\kappa }{1+\kappa }$, where the numeral 2 is associated with the power 2 of the Student’s *t* distribution, and the ratio $r=\frac{-2\kappa }{1+\kappa }$ is associated with a duality between the positive and negative domains of the coupling. The coupled entropy uses a generalization of the logarithmic function,

(A15) $$\ln_{\kappa }\left(x\right)\;\equiv \frac{1}{\kappa }\left(x^{\kappa }-1\right),\;\;x>0,$$

which provides a continuous set of functions with power. The coupled entropy aggregates the probabilities of a distribution using the generalized mean and translates this to the entropy domain using the generalized logarithm. Using the equiprobable for the sample probabilities, $p_{i}=\frac{1}{N}$, the coupled cross-entropy ‘score’ for the forecasted probabilities q for the event labels e is

(A16) $$S_{\kappa }\left(e,q\right)\;\equiv \frac{-2}{1+\kappa }\ln_{\left(\frac{-2\kappa }{1+\kappa }\right)}\left({\left(\frac{1}{N}\sum_{i=1}^{N}q_{i}^{\frac{-2\kappa }{1+\kappa }}\right)}^{\frac{-1-\kappa }{2\kappa }}\right)\equiv \frac{1}{\kappa }\left(\left(\frac{1}{N}\sum_{i=1}^{N}q_{i}^{\frac{-2\kappa }{1+\kappa }}\right)-1\right),$$

where $q_{i}$ is the probability of event $e_{i}$ which occurred. Thus, the coupled cross-entropy is a local scoring rule dependent only on the probabilities of the actual events.
